# Three-Dimensional Appearance and Physicochemical Properties of *Pleurotus eryngii* under Different Drying Methods

**DOI:** 10.3390/foods12101999

**Published:** 2023-05-15

**Authors:** Jun-Wen Bai, Yu-Chi Wang, Jian-Rong Cai, Lu Zhang, Yi Dai, Xiao-Yu Tian, Hong-Wei Xiao

**Affiliations:** 1 School of Food and Biological Engineering, Jiangsu University, Zhenjiang 212013, China; bjw@ujs.edu.cn (J.-W.B.);; 2 College of Engineering, China Agricultural University, Beijing 100083, China

**Keywords:** three-dimensional appearance, drying methods, quality, microstructure, *Pleurotus eryngii*

## Abstract

This study investigated the effects of different drying methods on the drying characteristics, three-dimensional (3D) appearance, color, total polysaccharide content (TPC), antioxidant activity, and microstructure of *Pleurotus eryngii* slices. The drying methods included hot air drying (HAD), infrared drying (ID), and microwave drying (MD). The results showed that the drying method and conditions significantly influenced the drying time, with MD having a significant advantage in reducing the drying time. The 3D appearance of *P. eryngii* slices was evaluated based on shrinkage and roughness as quantitative indexes, and the best appearance was obtained by hot air drying at 55 and 65 °C. HAD and ID at lower drying temperatures obtained better color, TPC, and antioxidant activity, but MD significantly damaged the color and nutritional quality of *P. eryngii*. The microstructure of dried *P. eryngii* slices was observed using scanning electron microscopy, and the results showed that drying methods and conditions had an obvious effect on the microstructure of *P. eryngii* slices. Scattered mycelia were clearly observed in *P. eryngii* samples dried by HAD and ID at lower drying temperatures, while high drying temperatures led to the cross-linking and aggregation of mycelia. This study offers scientific and technical support for choosing appropriate drying methods to achieve a desirable appearance and quality of dried *P. eryngii*.

## 1. Introduction

*Pleurotus eryngii*, also known as the king oyster mushroom, is called xingbaogu in China. As a member of the *Pleurotus species*, it is a valuable edible fungus that is rich in bioactive compounds, such as polysaccharides, proteins, and free amino acids. It also possesses numerous physiological functionalities, such as antimicrobial, antioxidant, hypolipidemic, hypoglycemic, and immune regulatory activities [1]. However, one of the challenges associated with using *P. eryngii* is its high moisture content, which can reach up to 90% on a wet basis. This makes fresh *P. eryngii* sensitive to bacterial growth and shortens its shelf life if not stored properly [2]. Therefore, there is an urgent need to implement processing techniques for *P. eryngii* in order to increase its shelf life and enhance its value-added potential throughout the industrial chain.

Drying is one of the most common processing methods for edible fungi [3,4]. The objective of drying is to reduce the moisture content, eliminate microorganisms, increase the soluble solids content, inhibit enzyme activity, extend the storage period, and enhance the value of commodities [5,6]. The most widely used drying method is hot air drying (HAD) due to its low cost, easy operation, and simple control [7,8]. In comparison, infrared drying (ID) can significantly shorten drying time and improve product quality, but its effectiveness can be influenced by infrared wavelengths, food surface porosity, and composition [9]. Although microwave drying (MD) is a fast and effective alternative technique, it has some drawbacks, such as a high initial cost, uneven heating, limited microwave penetration, and quality loss [10]. Appropriate drying methods and conditions can minimize nutrient and color degradation during the drying process of *P. eryngii*, reduce energy consumption, and ensure product quality.

The appearance of fruits and vegetables changes visibly during the drying process due to their porous tissue structure, resulting in shrinkage, curling, roughness, and collapse. With the advancement of computer vision (CV) technology, researchers use it to measure and analyze the changes in the appearance dimensions of materials during drying. Onwude et al. investigated the shrinkage of sweet potatoes using a combined digital and backscattering imaging system and found that shrinkage was affected by the product thickness, drying temperature, and drying time [11]. Pei et al. applied a camera to obtain the surface area and shrinkage of ginger slices, important quality parameters [12]. Li et al. observed the phenomenon of surface wrinkling of shiitake mushrooms during the drying process and obtained the ratio of the wrinkled surface area by region segmentation [13]. However, a single camera can only capture data on the projected area of the sample’s surface and cannot effectively measure changes in material thickness. To address this issue, Sampson et al. constructed a top and side dual-view computer vision system to measure the volume of apple slices [14]. Nonetheless, even with multiple cameras, the data obtained are still based on the analysis of two-dimensional (2D) projected images.

To obtain the three-dimensional (3D) appearance and shape changes of materials during the drying process, Cai et al. used the Kinect sensor to acquire depth and color images of potato slices and calculated the shrinkage and curling degree of the slices [15]. However, the accuracy limitations of the Kinect sensor [16] restrict its ability to distinguish changes within a range of 2 mm in practical applications. Bai et al. employed a binocular snapshot sensor to obtain point cloud images with a measurement accuracy of 0.035 mm [17]. They also calculated the shrinkage and height standard deviation (HSD) to evaluate the three-dimensional (3D) deformation, such as surface curl and flatness, of apple slices. Regarding edible fungi, particularly *P. eryngii*, the measurement and quantifiable representation indices for their 3D appearance changes are still limited in research.

This study aimed to investigate the effects of different drying methods and conditions on the drying characteristics, 3D appearance, physicochemical properties, and microstructure of dried *P. eryngii* slices. Furthermore, the study provides quantitative indexes for the analysis and evaluation of 3D appearance and offers technical support for selecting suitable drying methods for *P. eryngii*.

## 2. Materials and Methods

### 2.1. Samples and Chemicals

Fresh *P. eryngii* samples were purchased from a supermarket near Jiangsu University in Zhenjiang City, with an average wet basis moisture content of about 90.0 ± 1.0%. All the samples were stored in a refrigerator at 4 ± 1 °C and 90% relative humidity prior to the experiments. The analytical grade 2,2-diphenyl-1-picrylhydrazyl (DPPH), ethanol, concentrated sulfuric acid, phenol, and anhydrous glucose were purchased from Sinopharm Chemical Reagent Co., Ltd. (Shanghai, China).

### 2.2. Drying Experiments

Fresh *P. eryngii* samples were cut into circular slices approximately 5 mm in thickness with a diameter of 4.5 cm. The weight of raw samples was kept at 50.0 ± 0.5 g for all runs. The safe moisture content was set at 10% wet basis, according to the GB7096-2014 “National Food Safety Standard for Edible Fungi and Their Products”. The *P. eryngii* samples were periodically taken from the drying chamber for weighing and 3D point cloud collection until the samples reached the final moisture content during drying. All the drying experiments were conducted in triplicate.

Hot air drying (HAD): *P. eryngii* slices were dried under different temperatures of 55, 65, 75, and 85 °C. The air velocity was 3 m/s, and the relative humidity was 20%. The samples were laid flat on trays in a hot air dryer, which was self-developed by Jiangsu University [18].

Infrared drying (ID): Samples were dried under different temperatures of 60, 65, 75, and 85 °C. The infrared equipment was a Hauswirt electric infrared oven (I7, Hauswirt, China). No airflow was used in the infrared oven, and the distance between the samples and the infrared source was approximately 15 cm.

Microwave drying (MD): Drying experiments were carried out in a domestic digital microwave oven (P70d2otl, Galanz, China), with a maximum power output capacity of 700 W at 2450 MHz. The microwave oven has a capability to operate at four different microwave output powers (119, 157, 196, and 280 W), with a measurement accuracy of ±10 W. Processing time and microwave output power were adjusted using the digital control on the microwave oven. The *P. eryngii* slices were placed in a single layer on a rotating glass plate in the oven to ensure uniform heating.

### 2.3. Drying Characteristics

The initial moisture content of *P. eryngii* slices was measured using a quick moisture analyzer (DHS-16, Lichen instrument technology Co., Ltd., Shanghai, China). The moisture ratio was calculated using Equations (1) and (2) [19,20];

(1) $$MR=\frac{M_{t}-M_{e}}{M_{0}-M_{e}}$$

where *M*_0_ is the initial dry basis moisture content; *M_t_* is the dry basis moisture content at the drying time; *MR* is the moisture ratio; *M_e_* is the equilibrium moisture content. As in the equilibrium moisture content, *M_e_* is much smaller than *M_t_* and *M*_0_, it can generally be ignored [21]. Therefore, the calculation of *MR* can be simplified as

(2) $$MR=\frac{M_{t}}{M_{0}}$$

### 2.4. Point Cloud Data Acquisition

A 3D point cloud acquisition platform developed by our research group [17,18] was used in this study. The platform uses a binocular snapshot Gocator3210 sensor (LMI, Canada) with a detection accuracy of 0.035 mm. Before data collection, the datum plane was calibrated, and then point cloud images of the *P. eryngii* slices were successively captured during the drying process.

### 2.5. Three-Dimensional Appearance Evaluation Index

The 3D point cloud processing software Cloud Compare was used to process the acquired images, including background removal, point cloud filtering, surface reconstruction, and surface smoothing [19]. After the point cloud processing, the images were subjected to feature extraction, and two quantitative indexes (shrinkage and roughness) were selected to evaluate the 3D appearance of the *P. eryngii* slices during drying.

#### 2.5.1. Shrinkage

The surface model was composed of tens of thousands of triangles. First, the distance between two points was calculated using Euclid’s formula to obtain the three side lengths of each triangle. For example, the distance between the point *p*_1_ (*x*_1_, *y*_1_, *z*_1_) and *p*_2_ (*x*_2_, *y*_2_, *z*_2_) can be calculated using Equation (3). Then, the area of each triangle was calculated using Helen’s formula, as shown in Equation (4), and the sum of the area of all triangles was calculated, which was the surface area. The shrinkage of the *P. eryngii* slices during drying can be calculated by the change in surface area at different drying time points using Equation (5). The specific formulas are as follows:

(3) $$d_{p_{1}p_{2}}=\sqrt{{\left(x_{1}-x_{2}\right)}^{2}+{\left(y_{1}-y_{2}\right)}^{2}+{\left(z_{1}-z_{2}\right)}^{2}}$$

(4) $$S_{ABC}=\sqrt{p(p-d_{AB})(p-d_{AC})(p-d_{BC})}$$

(5) $$Shrinkage=\frac{S_{0}-S_{t}}{S_{0}}$$

where *d*_*p*1*p*2_ is the distance between the two points of *p*_1_ and *p*_2_, *S_ABC_* is the area of the triangle *ABC*, and *p* is half of the perimeter of the triangle *ABC*. *S*_0_ is the surface area of the sample before drying, and *S_t_* is the surface area of the sample during drying.

#### 2.5.2. Roughness

During the drying process, the surface of the material becomes convex and uneven, so roughness was selected to evaluate the smoothness of the material surface. The principle of calculation is as follows: for each point in the point cloud data, the radius of the input parameter R is used to find the adjacent points in this range and fit the least-squares plane. The distance d of the point to the least-squares plane is calculated as the roughness. If the least-squares plane cannot be fitted to a point in the point cloud data, the roughness of the point is defined as null. According to the experimental results, the best input radius was set to 2 mm.

### 2.6. Color Measurement

The color of the dried samples was measured using a colorimeter (SC-10, Shenzhen 3nh Technology Co., Ltd., Shenzhen, China). The color coordinates included L* (lightness, 0~100), a* (redness, −60~60), and b* (yellowness, −60~60). Fresh *P. eryngii* slices were used as the control, and the total color difference (Δ*E*) was calculated according to Equation (6) [22]. The color parameters of each sample were measured 9 times and averaged:

(6) $$\Delta E=\sqrt{{\left(L^{*}-L_{0}^{*}\right)}^{2}+{\left(a^{*}-a_{0}^{*}\right)}^{2}+{\left(b^{*}-b_{0}^{*}\right)}^{2}}$$

where $L_{0}^{*},\;a_{0}^{*},\;b_{0}^{*}$ are the color parameters of fresh *P. eryngii* slices, and L*, a*, b* are the color parameters of dried *P. eryngii* slices.

### 2.7. Determination of Total Polysaccharide Content (TPC)

To determine the crude polysaccharide content, we followed the NY/T 1676-2008 method for determining the crude polysaccharide content in edible fungi. The total polysaccharide content of the extraction was measured using the phenol–sulfuric acid method, as described by Su et al. [23] and Zhu et al. [24]. The crude polysaccharides from *P. eryngii* slices were extracted using ultrasonic-assisted extraction (YM-040S, Fangao Microelectronics Co., Ltd., Shenzhen, China). The results were measured using dry mass, and all units were expressed as mg/g. Each experiment was repeated three times.

### 2.8. Determination of DPPH Radical Scavenging Activity

The sample was homogenized with 1:40 (*w*/*v*) ethanol to obtain the extract, which was used for scavenging DPPH free radical activity. After filtering, the mixture was centrifuged (4000 rpm, 10 min), and then the supernatant was collected. The following measurements were conducted following the method described by Liu et al. [25]. A suitable volume of the *P. eryngii* slices extract was mixed with 2 mL of DPPH ethanol solution and placed in the dark at 37 °C for 30 min. The absorbance of the reaction solution was measured at 517 nm, and a blank control was established using ethanol instead of the sample solution in the DPPH ethanol mixture. The DPPH radical scavenging activity of *P. eryngii* slices was calculated using Equation (7) as described by Bechlin et al. [26]. Each group of samples was repeated three times.

(7) $$DPPH\;radical\;scavenging\;activity=(1-\frac{A}{A_{0}})\times 100\%$$

where *A* is the absorbance value of dried samples, and *A_0_* is the absorbance value of the control.

### 2.9. Microstructure Analysis

Scanning electron microscopy (S-3400N, Hitachi Co., Ltd., Tokyo, Japan), at an accelerating voltage of 15 kV vacuum, was used to examine the microstructure of the dried *P. eryngii* slices. Each sample was cut into blocks with a cross-section of 5 mm × 5 mm, and microstructure images of 200× and 1000× were obtained after sample preparation and gold spraying.

### 2.10. Statistical Analysis

All experiments were conducted in triplicate, and data were presented in the form of mean ± standard deviation values. The experimental data of the drying process were analyzed using origin 2018 and Cloud Compare software. The results were evaluated using one-way analysis of variance (ANOVA) followed by Duncan’s new multiple range test (SPSS 25.0 software). Differences were considered significant at *p* < 0.05.

## 3. Results and Discussions

### 3.1. Drying Curves

**Figure 1:**
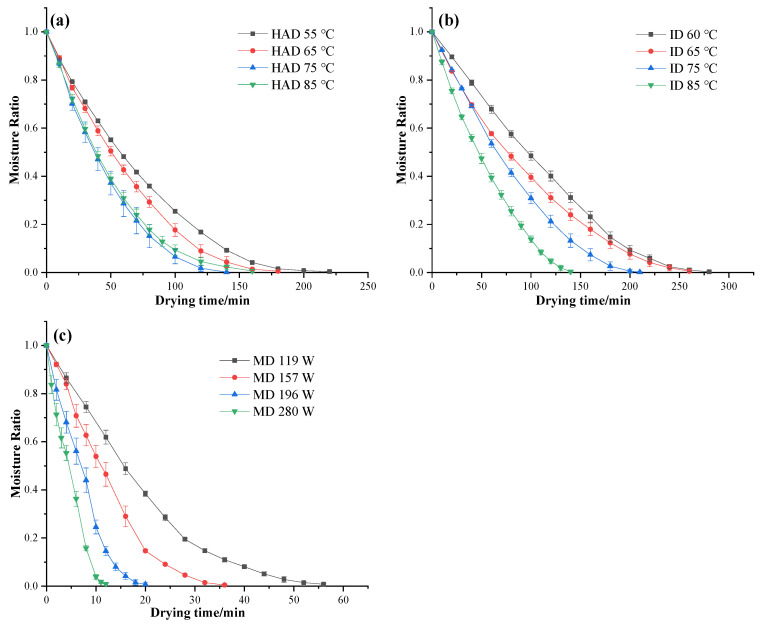
Drying curves of *P. eryngii* slices under different drying conditions. (**a**) Hot air drying, (**b**) infrared drying, (**c**) microwave drying.

Figure 1 shows the moisture ratio (MR) curves versus drying time of *P. eryngii* slices under different drying conditions. From Figure 1a, it can be seen that the time required to reduce the moisture from the initial moisture content to the desired moisture content was approximately 220, 180, 140, and 160 min, respectively, for hot air temperatures of 55, 65, 75, and 85 °C. It is worth noting that the drying time of *P. eryngii* slices at 85 °C is about 20 min longer than that at 75 °C. This could be attributed to the high drying temperature accelerating the drying of the surface, causing surface hardening, which hindered the transfer of moisture inside the samples [27]. Demirel and Turhan also reported that the effective moisture diffusivity of banana slices at 70 °C was lower than at 60 °C due to starch gelatinization and surface hardening [28].

From Figure 1b, it can be observed that the drying time of *P. eryngii* slices dried by ID was approximately 280, 260, 210, and 140 min at drying temperatures of 60, 65, 75, and 85 °C, respectively. The drying time decreased by 50% with the ID temperature increasing from 60 to 85 °C. Several studies have shown that ID can reduce the drying time compared to HAD. For example, Cao et al. [29] reported that the drying time of ID was reduced by 38.27%, 34.62%, and 30.56% compared to HAD under the corresponding temperatures of 60, 70, and 80 °C, respectively. However, the results of this study showed the opposite. Nowak et al. reported that the drying time of ID was dependent on emitter–sample distance, air velocity, air temperature, and infrared radiation intensity [30,31]. The difference in results from Cao et al. could be due to the absence of airflow in the ID equipment and the relatively long distance from irradiation.

For microwave drying (Figure 1c), the drying time was about 56, 36, 20, and 12 min for 119, 157, 196, and 280 W, respectively. It is evident that MW resulted in a considerable reduction in drying time compared to HAD and ID. Albanese et al. also reported that using MW for apricots reduced the drying time by more than 25% when compared to HAD [32]. Therefore, MW has significant advantages in reducing the drying time of *P. eryngii* slices. However, the appearance and quality characteristics of the product under different drying methods still require further investigation.

### 3.2. Three-Dimensional Appearance Characterization

**Figure 2:**
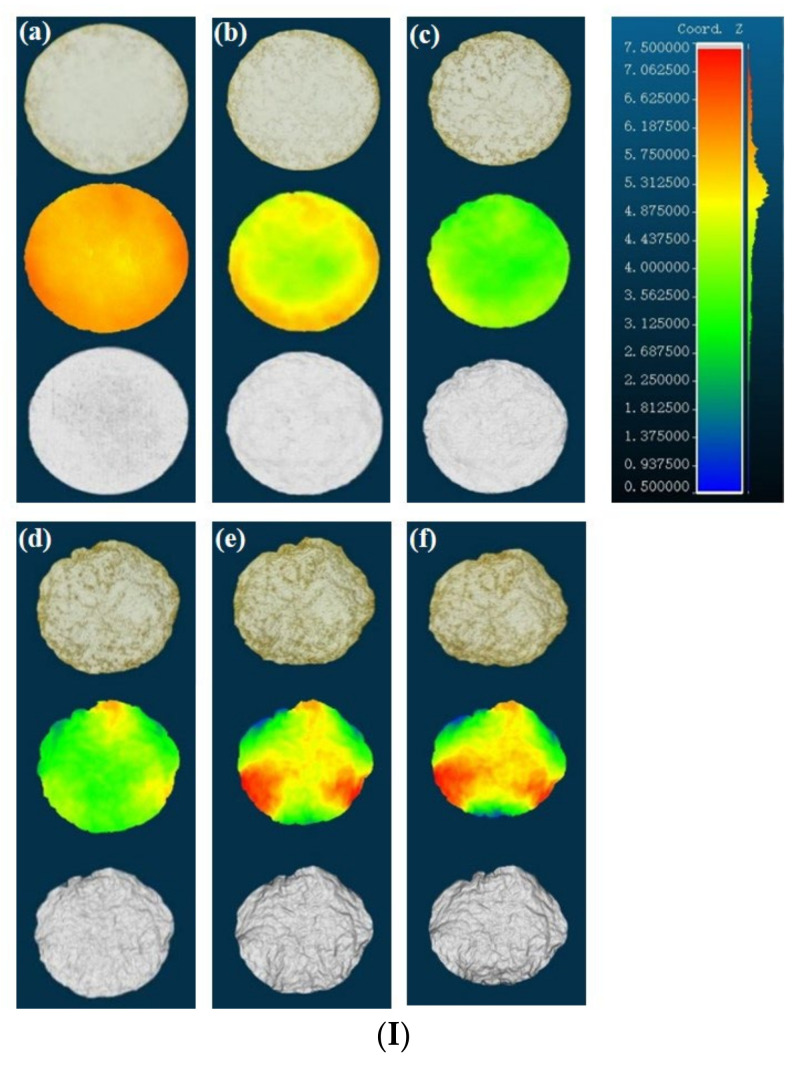

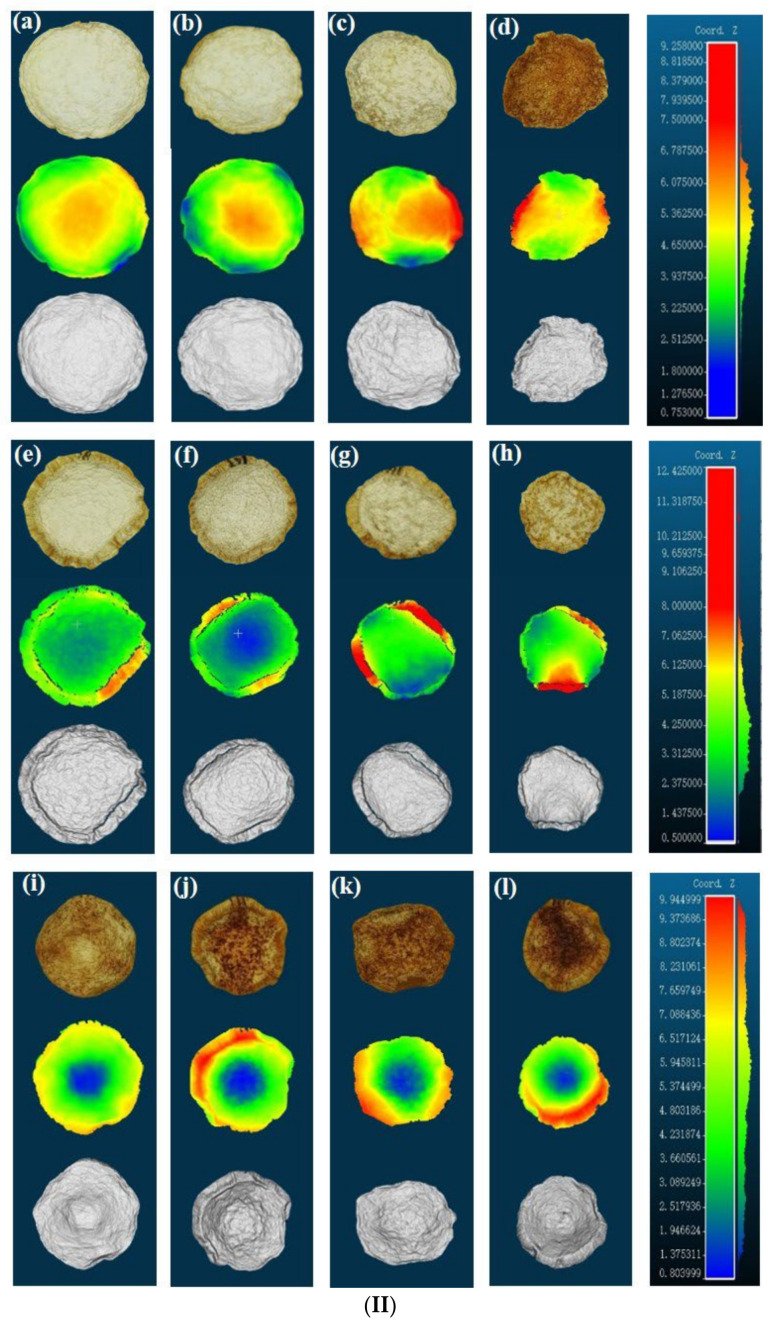
(**I**) Time-varying appearance images of one *P. eryngii* slice during drying. (**Ia**–**If**) represent *P. eryngii* slices dried at 75 °C, 20% RH, 3 m/s for 0, 30, 60, 90, 120, 140 min under hot air drying, respectively. The three images from top to bottom in each column represent color physical images, height-distributed images, and 3D reconstructed images. (**II**) Three-dimensional appearance images of dried *P. eryngii* slices under different drying methods. (**IIa**–**IId**) Hot air drying at 55, 65, 75, and 85 °C. (**IIe**–**IIh**) Infrared drying at 60, 65, 75, and 85 °C. (**IIi**–**IIl**) Microwaves drying at 119, 157, 196, 280 W. The three images from top to bottom in each column represent color physical images, height-distributed images, and 3D reconstructed images of *P. eryngii* slices.

The time-varying appearance images of a *P. eryngii* slice at 75 °C under HAD are displayed in Figure 2I. In each column, the three images from top to bottom represent a color physical image, a height-distributed image, and a 3D reconstructed image. The height distribution of the *P. eryngii* slice in Figure 2 is depicted using pseudo-color images, where the color from blue to red indicates that the height value of the pixels on the material changed from small to large. The 3D reconstructed images, created by the greedy projection algorithm, accurately reproduce the 3D appearance changes of the *P. eryngii* slice during drying [33]. According to the height-distributed images, the thickness of the *P. eryngii* slices was relatively uniform at the beginning of drying, and the only morphological change was a decrease in thickness. After 30 min of drying, the central part of the sample began to thin and develop a concave shape. After 120 min, the edges of the sample curled significantly, and the surface became rough with an increase in shrinkage.

The appearance images of the dried *P. eryngii* slices under different drying methods are shown in Figure 2II. It was found that the appearance of the *P. eryngii* slices had significant curling, shrinkage, and browning after the drying process. Moreover, the appearance of the dried *P. eryngii* slices varied greatly under different methods and conditions. The appearance of microwave-dried *P. eryngii* slices changed most seriously, with the color and shape differing greatly from the initial state.

Figure 2(IIa–IId) shows the appearance of *P. eryngii* slices under HAD at 55, 65, 75, and 85 °C. The figure illustrates that at lower temperatures (55 °C and 65 °C), *P. eryngii* slices dried by HAD had a relatively flat appearance. However, when the drying temperature was increased, the surface of *P. eryngii* slices underwent significant browning, severe curling, and shrinkage. In addition, brown spots and bumps also appeared on the surface of the samples. The appearance of *P. eryngii* slices under ID at 60, 65, 75, and 85 °C is shown in Figure 2(IIe–IIh). The images show that as the drying temperature increased, the samples with ID did not shrink uniformly from all directions towards the center but rather sank downwards in the middle and curled upwards around the edges. Due to uneven heating during MD, some parts of the *P. eryngii* slices were burnt, resulting in pits or bumps in the middle, along with a burning smell (Figure 2(IIi–IIl)). Additionally, the appearance of the samples shrank significantly after microwave drying, and the surface exhibited obvious browning. To address the challenge of controlling material temperatures during the later stages of MD, a common practice involves utilizing microwave predrying as a fast initial step, followed by traditional hot air convection drying. This combined method is effective in achieving a balanced drying rate while maintaining product quality [34]. The corresponding 3D reconstructions and height distribution images vividly show the appearance and color changes of *P. eryngii* slices under different drying methods.

### 3.3. Shrinkage

**Figure 3:**
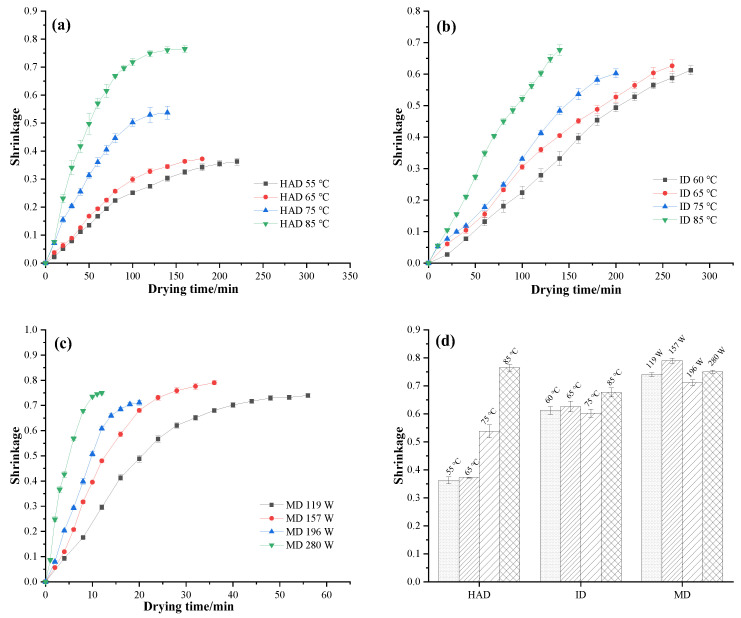
Shrinkage of *P. eryngii* slices during drying under different drying conditions, such as (**a**) hot air drying, (**b**) infrared drying, and (**c**) microwave drying, (**d**) the shrinkage of *P. eryngii* at the end point of drying.

Figure 3 depicts the shrinkage curves of *P. eryngii* under different drying conditions. The curves indicate that the shrinkage of *P. eryngii* slices occurs mainly during the early drying stage and gradually slows down in the later drying stage. Previous research showed that shrinkage at the initial stage of drying is approximately equal to the volume of moisture lost. In the middle and late stages, as the “skeleton” becomes fixed, the shrinkage slows down [35]. Figure 3a shows that the shrinkage of *P. eryngii* samples dried by HAD were 0.3625, 0.3718, 0.5372, and 0.7647 at drying temperatures of 55, 65, 75, and 85 °C, respectively. As the drying temperature increased, the shrinkage rate of *P. eryngii* significantly increased, indicating that high temperatures exacerbate the shrinkage of dried samples. This phenomenon was caused by accelerated water removal under high temperatures, which led to more stress in the matrix of the food material. Wang et al. observed that the HAD process on jujube slices caused serious volume shrinkage due to the high temperatures involved [36]. Udomkun et al. also found that an increase in drying temperature from 50 to 80 °C led to an increase in papaya shrinkage at the same final moisture content [37].

As shown in Figure 3b, the shrinkage of ID *P. eryngii* samples was 0.6122, 0.6264, 0.6024, and 0.6767 at drying temperatures of 60, 65, 75, and 85 °C, respectively. The shrinkage rate increased with increasing drying time. Shrinkage at 75 °C was significantly lower (*p* < 0.05) than that at 60 and 65 °C. This may be because the higher temperatures during case hardening hindered further shrinkage. It is worth noting that, compared to HAD dried samples, the shrinkage of ID samples was more obvious. Kaveh et al. also found that green beans dried by infrared drying had greater shrinkage than those dried by hot air drying [38]. The shrinkage of *P. eryngii* samples dried by MD ranged from 0.6712 to 0.7760. Compared to the other two drying methods, the shrinkage of *P. eryngii* by MD was more severe. This may be due to the fact that microwaves can directly heat the interior of the sample, which may increase its internal temperature and produce water vapor, providing an additional driving force for water migration, thus increasing the drying rate and accelerating shrinkage [39]. Rokhbin and Azadbakht found that any rise in microwave power (from 90 to 900 W) led to an intensification of orange slice shrinkage [40]. However, in this experiment, the shrinkage rate did not increase continuously with the increase in microwave power. According to the report by Mahiuddin et al., tissue structure is an important factor affecting contraction behavior [41], so the difference between the obtained data and previous studies might be due to the difference in the tissue structure of different plant species.

### 3.4. Roughness

**Figure 4:**
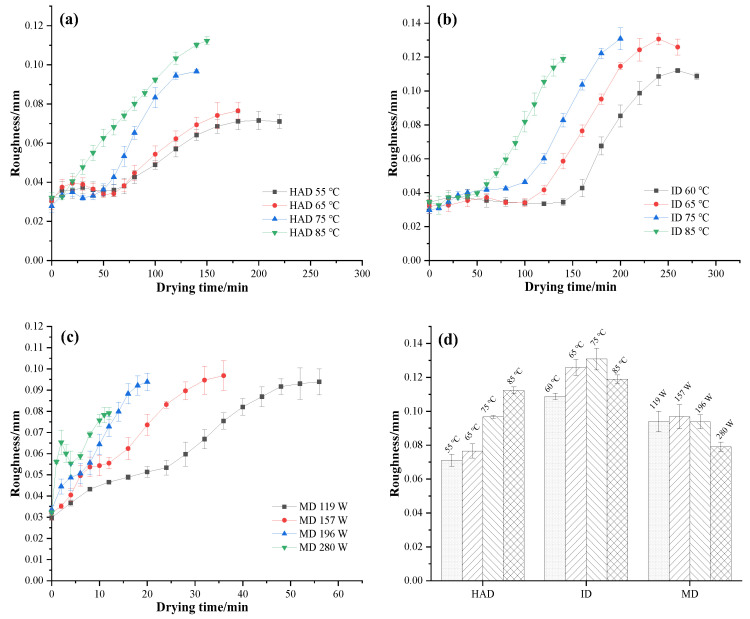
Roughness of *P. eryngii* slices during drying under different drying conditions, such as (**a**) hot air drying, (**b**) infrared drying, and (**c**) microwave drying, (**d**) the roughness of *P. eryngii* at the end point of drying.

The effects of drying conditions on the roughness of *P. eryngii* are shown in Figure 4. It is evident that the surface roughness increases with the extension of drying time. Moreover, the roughness of dried *P. eryngii* also increases with an increase in the HAD temperature from 55 °C to 85 °C (Figure 4a). This phenomenon can be attributed to the uneven distribution of moisture and the surface stress formed by the heat effect, resulting in an uneven appearance. Furthermore, polysaccharides tend to depolymerize into small molecules of polysaccharides or monosaccharides under higher temperatures, increasing their hygroscopicity and leading to mycelium aggregation, which can cause an uneven surface texture.

During infrared drying, the roughness of *P. eryngii* increased gradually over the drying time, showing a similar trend as that of hot air drying. However, the roughness of *P. eryngii* dried by ID was noticeably higher than that of samples dried by HAD. Yang et al. noted that the uneven distribution of water diffusion, as well as the collapse of the cellular structure on the surface, ultimately resulted in uneven structures, which may be attributed to the limited depth of infrared radiation [42].

After microwave drying, the roughness values of *P. eryngii* slices at the end point of drying ranged from 0.0782 to 0.0947. The roughness of samples dried by MD was lower than that of samples dried by ID, but higher than those dried at 75 °C and 85 °C using HAD. Considering all three drying methods and conditions, the roughness of *P. eryngii* was lowest when dried with low-temperature HAD, resulting in the smoothest surface of the dried samples. It was reported that the larger difference between the vapor pressure of water on the surface of the mushrooms and the bulk air partial pressure of water vapor resulted in a larger driving force for drying, leading to more rapid dehydration, which promoted wrinkling [13]. Under low-temperature drying conditions, the driving force for the drying of *P. eryngii* is relatively weak. This facilitates the release of stress caused by the uneven distribution of moisture on the surface of the material, thereby reducing wrinkling and shrinkage on its surface.

### 3.5. Color

Color is one of the most important sensory attributes of edible mushrooms, as it influences the customer’s perception and purchasing power. The color values of fresh and dried *P. eryngii* slices using different drying methods are presented in Table 1. The L* values of dried samples were significantly (*p* < 0.05) lower, while the values of a* and b* were significantly higher (*p* < 0.05) than the values of the fresh *P. eryngii* slices. Similar results were also reported in shiitake mushrooms [43]. It is generally believed that the observer can detect the color difference clearly when the Δ*E* is greater than 5 [44]. The ∆*E* of the dried *P. eryngii* slices obtained by different drying methods were higher than 24, indicating that the drying process significantly changed the color of *P. eryngii* slices.

By observing Figure 2(II) and Table 1, it can be seen that the color of the sample is better at lower drying temperatures. This can be explained by the fact that enzymatic browning reactions and nonenzymatic browning reactions were significantly attenuated at lower temperatures [42]. Gouzi et al. [45] found that the polyphenol oxidase (PPO) activities of the mushroom *Agaricus bisporus* were still retained by approximately 15% at 60 °C after 30 min of heat treatment. Although the drying temperature is higher than the temperature of PPO enzyme inactivation, the internal temperature of the material is usually less than the drying temperature in most of the drying time due to the effect of moisture evaporation and heat absorption [46]. It was also reported that both nonenzymatic browning and enzymatic browning take place during the drying process of button mushrooms, but the effect of enzymatic browning is more significant [47]. MD-dried samples presented significantly lower L* values than the HAD and ID samples. Meanwhile, MD also particularly influenced the a* and b* values, resulting in the highest value of ∆*E* (50.94). This might be due to the phenomenon of coke spot caused by the excessive heating of materials in the MD process. Therefore, using color as the evaluation index, it can be concluded that the HAD and ID methods produce acceptable dried *P. eryngii*, whereas the MD method is not recommended.

### 3.6. TPC

The TPC of fresh and dried *P. eryngii* slices under different conditions is listed in Table 1. The TPC of all dried samples was lower than that of the fresh samples (48.37 ± 0.75 mg/g). This can be attributed to the thermal decomposition of polysaccharides and the Maillard reaction during heat treatment. By comparing the TPC of dried *P. eryngii* slices under three drying methods, it was found that the retention of total polysaccharides by HAD and ID had little difference. However, MD-dried *P. eryngii* slices had the lowest retention rate of total polysaccharides. This result may be due to the limited availability of water at the final stages of the MD process, which can cause the temperature of the material to easily rise to a level that accelerates the degradation of polysaccharides [48,49]. The TPC of *P. eryngii* slices decreased with increasing microwave power, and the lowest TPC was 28.82 ± 0.50 mg/g.

### 3.7. DPPH Radical Scavenging Activity

The values of DPPH radical scavenging activity of *P. eryngii* slices under different drying methods and conditions are shown in Table 1. According to the DPPH assay, the antioxidant activity of the fresh sample was significantly stronger than that of the dried samples (*p* < 0.05). Similarly, Gasecka et al. found that DPPH scavenging activity in fresh samples was significantly higher than that in dried samples [50]. Normally, the antioxidant activity of plant materials depends on their content of bioactive components. Drying is a thermal process that results in a decrease in the content of heat-sensitive substances with antioxidant activity.

For the HAD and MD processes, the DPPH radical scavenging activity of *P. eryngii* significantly decreased with increasing drying temperatures and microwave power. Different from the above two drying methods, the DPPH radical scavenging activity of *P. eryngii* slices dried by ID increased with the increase in drying temperature. Infrared drying at 85 °C obtained the highest antioxidant activity (46.55%), while microwave drying at 280 W showed the lowest antioxidant activity (25.89%). In contrast to our study, Ren et al. found that the antioxidant activity of *Coprinus comatus* dried by microwave drying was superior to that of hot air and infrared drying [51]. This difference in results may be attributed to the significantly higher microwave power used in Ren et al.’s study, which was set at 700 W, compared to the range of drying conditions used in our study. This high microwave power setting may have greatly reduced the drying time, thereby preserving more antioxidant compounds in the dried samples. The discrepancy in microwave power used in the two studies may have led to the difference in the antioxidant activity observed in the dried mushroom products.

Overall, the differences in the DPPH radical scavenging activity of dried samples under different drying methods and conditions may be due to the effects of these factors on the preservation of antioxidants in the samples. The retention of the antioxidants in the dried samples depends on both the processing temperature and drying time. Higher drying temperatures may not help to preserve the antioxidant compounds, but high temperatures can significantly shorten the drying time, thus reducing the exposure time of materials to oxygen and thermal effects. Wu et al. also discovered that reducing the drying time improved the DPPH radical scavenging activity of the dried samples [52]. The antioxidant activity of dried samples is determined by the combination of drying methods and conditions; therefore, selecting the appropriate drying methods and conditions is crucial for improving the antioxidant activity of dried products.

### 3.8. Microstructure

**Figure 5:**
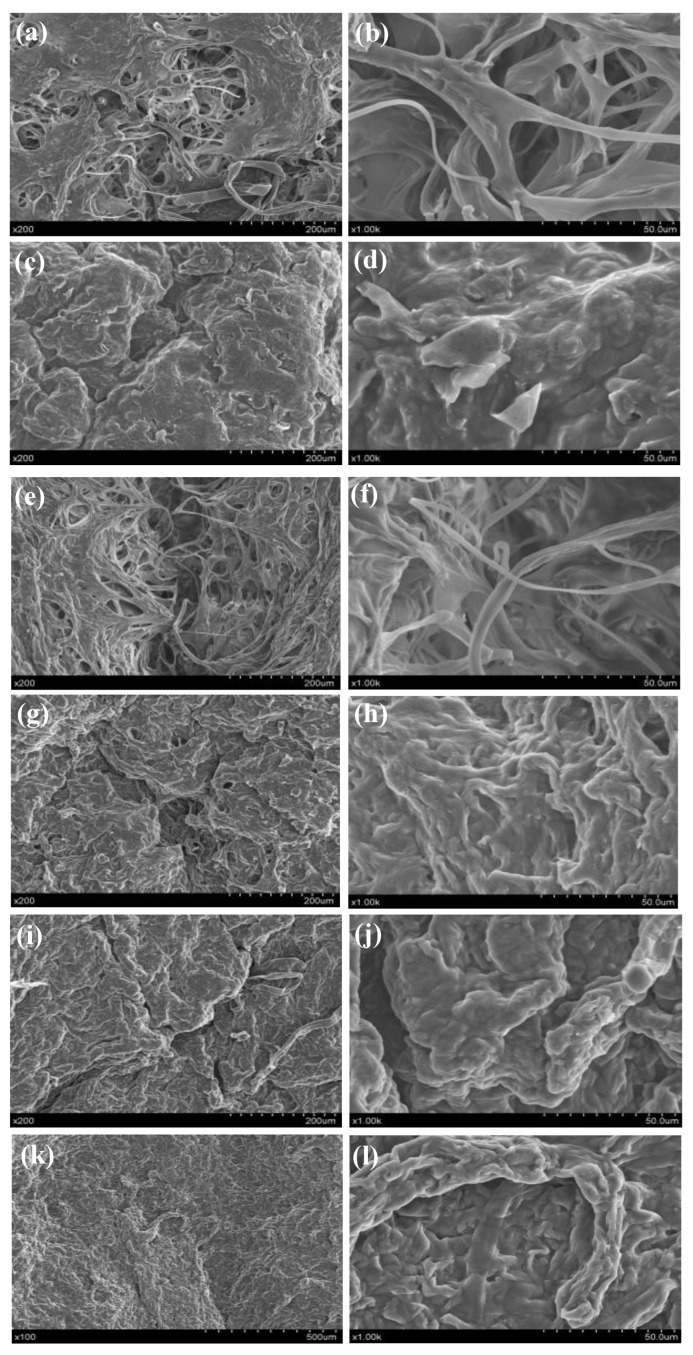
Microstructure of dried *P. eryngii* slices under different drying conditions at different magnifications. (**a**,**b**) Hot air drying at 65 °C; (**c**,**d**) hot air drying at 85 °C; (**e**,**f**) infrared drying at 65 °C; (**g**,**h**) infrared drying at 85 °C; (**i**,**j**) microwave drying at 157 W; (**k**,**l**) microwave drying at 280 W.

The microstructures of dried *P. eryngii* slices under different drying conditions (Figure 5) were observed using SEM. The images showed that the drying methods and conditions had an obvious effect on the tissue structure of *P. eryngii* slices. When the samples were dried by HAD at 65 °C (Figure 5a,b) and ID at 65 °C (Figure 5e,f), their loose porous structure was clearly visible, and the individual mycelium could be easily distinguished. When the samples were dried by microwave (Figure 5i–l), hot air at 85 °C (Figure 5c,d), and infrared at 85 °C (Figure 5g,h), the mycelium was completely gathered and cross-linked. This could be due to the depolymerization of mycelium cell wall polysaccharides under high temperatures, which could damage the structure of mycelium. Additionally, the Maillard reaction may also cause the loss of polysaccharides and the cross-link reaction with proteins [51,53]. By matching Figure 5 with the appearance images in Figure 2, it can be concluded that the appearance changes of *P. eryngii* were related to the microstructure.

## 4. Conclusions

The effects of different drying methods and conditions on the drying characteristics, 3D appearance, physicochemical properties, and microstructure of *P. eryngii* slices were investigated. The drying method and conditions significantly influenced the drying time of *P. eryngii*, and microwave drying had a significant advantage in reducing drying time. The results showed that the shrinkage and roughness of *P. eryngii* slices gradually increased during drying, which was significantly related to the drying method and conditions. The best appearance of *P. eryngii* was obtained by hot air drying at 55 and 65 °C. The color, total polysaccharide content, antioxidant activity, and microstructure of dried *P. eryngii* slices were also analyzed. HAD and ID at lower drying temperatures achieved better product quality, but MD significantly damaged the color and nutritional quality of *P. eryngii*, so MD cannot be recommended. The microstructures of dried *P. eryngii* slices were observed using SEM. The observation of microstructures is helpful to understand the appearance and quality changes of *P. eryngii* during drying.

## Figures and Tables

**Table 1:** Physicochemical properties of P. eryngii slices under different drying conditions.

Drying Condition		L*	a*	b*	Δ*E*	TPC (mg/g)	DPPH Radical Scavenging Activity (%)
Fresh	——	85.62 ± 0.33 ^a^	3.95 ± 0.19 ^h^	12.85 ± 0.39 ^f^	——	48.37 ± 0.75 ^a^	68.87 ± 1.03 ^a^
HAD	55 °C	76.15 ± 4.87 ^b^	15.63 ± 0.72 ^f^	31.74 ± 2.27 ^c^	24.40 ± 1.15 ^g^	30.70 ± 0.32 ^b,c^	43.91 ± 2.04 ^b,c^
	65 °C	71.46 ± 5.86 ^c,d^	14.68 ± 0.66 ^g^	28.54 ± 2.04 ^d^	24.71 ± 2.23 ^g^	30.88 ± 0.15 ^b^	40.30 ± 2.08 ^d^
	75 °C	66.21 ± 4.53 ^e,f^	16.20 ± 0.59 ^f^	31.74 ± 1.52 ^c^	29.99 ± 2.76 ^f^	30.57 ± 0.08 ^b,c^	32.80 ± 0.15 ^e^
	85 °C	50.19 ± 1.81 ^h^	21.83 ± 0.80 ^b,c^	38.34 ± 2.00 ^b^	47.25 ± 0.31 ^c^	30.50 ± 0.1 ^b,c^	32.69 ± 0.65 ^e^
ID	60 °C	68.89 ± 1.97 ^d,e^	16.18 ± 0.29 ^f^	32.01 ± 0.66 ^c^	28.29 ± 0.95 ^f^	30.55 ± 0.13 ^b,c^	42.24 ± 0.70 ^d^
	65 °C	66.08 ± 1.22 ^e,f^	16.06 ± 0.52 ^f^	31.16 ± 1.07 ^c^	29.40 ± 1.51 ^f^	30.63 ± 0.20 ^b,c^	43.87 ± 0.71 ^b,c^
	75 °C	74.18 ± 3.09 ^b,c^	16.05 ± 0.22 ^f^	32..30 ± 0.63 ^c^	25.77 ± 1.21 ^g^	30.32 ± 0.3 ^b,c^	45.21 ± 0.75 ^b^
	85 °C	63.92 ± 1.09 ^f^	19.97 ± 0.53 ^e^	38.46 ± 1.41 ^b^	37.20 ± 1.68 ^e^	30.00 ± 0.94 ^b,c,d^	46.55 ± 0.24 ^b^
MD	119 W	57.26 ± 2.90 ^g^	22.73 ± 0.52 ^a,b^	42.42 ± 0.95 ^a^	45.13 ± 1.93 ^d^	29.90 ± 0.92 ^b,c,d^	40.25 ± 3.11 ^c^
	157 W	37.87 ± 2.65 ^i^	20.61 ± 2.15 ^d,e^	25.69 ± 3.65 ^e^	52.41 ± 1.04 ^a^	29.95 ± 0.90 ^b,c,d^	39.20 ± 2.30 ^d^
	196 W	47.09 ± 4.23 ^h^	23.22 ± 1.37 ^a^	36.58 ± 4.27 ^b^	49.56 ± 1.14 ^b^	29.32 ± 0.07 ^c,d^	32.29 ± 1.95 ^e^
	280 W	40.77 ± 3.63 ^i^	21.39 ± 1.23 ^c,d^	28.97 ± 3.29 ^d^	50.94 ± 2.32 ^a,b^	28.82 ± 0.50 ^d^	25.89 ± 0.90 ^f^

Note: The data are presented as the mean ± standard deviation for three replicates. Different letters (a, b, c, d, e, f, g, h, and i) in the same column for each parameter indicate significant differences.

## Data Availability

The data that support the findings of this study are available from the corresponding author upon reasonable request.
